# Short-term acute outcomes by clinical and socioeconomic characteristics in adults with SARS-CoV-2: a population-based cohort study focused on the first two years of the COVID-19 pandemic

**DOI:** 10.1186/s13690-025-01537-z

**Published:** 2025-03-24

**Authors:** Alice Corsaro, Federico Banchelli, Rossella Buttazzi, Enrico Ricchizzi, Carlo Gagliotti, Elisa Fabbri, Elisa Gentilotti, Maurizia Rolli, Evelina Tacconelli, Maria Luisa Moro, Nicola Caranci, Elena Berti

**Affiliations:** 1 Department of innovation in healthcare and social services, Emilia-Romagna Region, Bologna, Italy; 2 Public Health Department, Local Health Authority of Parma, Parma, Italy; 3 Regional Health and Social Care Agency, Emilia-Romagna Region, Bologna, Italy; 4 Department of Diagnostics and Public Health, University of Verona, Verona, Italy

**Keywords:** SARS-CoV-2, COVID-19, Acute phase, Short-term severe outcomes, Hospitalization, Mortality, Acute COVID-19 severity, Population-based cohort, Socioeconomic characteristics, ORCHESTRA project

## Abstract

**Background:**

The COVID-19 pandemic disproportionately affected vulnerable populations in terms of comorbidity and socioeconomic disadvantage, both between and within countries. This retrospective population-based cohort study is part of the Horizon 2020 ORCHESTRA project, was conducted in the Emilia-Romagna (E-R) Region, and aimed to investigate the risk of hospitalization, disease severity and all-cause mortality during the 30 days following SARS-CoV-2 infection.

**Methods:**

All adult positive cases notified in E-R from 2020 to 2022 were included. Poisson regression with robust standard error was used to estimate risk ratios for the three outcomes, stratified by sex, pandemic period and adjusted for age, citizenship, deprivation index, risk of hospitalization and death score (RHDS), and vaccination status. Data sources were regional healthcare databases. Supplementary analyses considered citizenship in relation to duration of residency in E-R or aggregated in areas of origin.

**Results:**

During the first two years of the pandemic 859,653 E-R residents tested positive for SARS-CoV-2 (47.8% males); 9.6% of them were citizens from high migratory pressure countries (HMPCs). The risk of severe outcomes increased steeply with age, especially in males. RHDS predicted worse outcomes in both sexes while vaccination showed a strong protective effect against all outcomes of acute infection (i.e., recent vaccination was 85% more protective against in-hospital severe disease in both sexes). Immigrants from HPMCs, especially females, showed a higher risk of hospitalization and in-hospital severe disease, in particular those who arrived within 5 years ago from the infection (RR for hospitalization = 1.92, 95%CI = 1.76-2.00 for males, and RR = 2.40, 95%CI = 2.23–2.59 for females), whereas the risk of all-cause mortality was lower compared to residents from low migratory pressure countries (LMPCs) that showed a RR for females of 0.73 (95%CI = 0.59–0.90).

**Conclusions:**

The results provided an overall view of course of acute COVID-19 outcomes in E-R and allowed the risk associated with clinical, demographic, and social characteristics to be measured. The findings suggest that, although national and regional public health policies have helped to mitigate the impact of the pandemic in the general population, inequalities in outcomes among persons with comorbidities and social disadvantages remain. Improvements in the appropriateness, effectiveness and equity of public health strategies are needed.

**Supplementary Information:**

The online version contains supplementary material available at 10.1186/s13690-025-01537-z.

**Table:** 

Text box 1. Contributions to the literature
• The spread and health outcomes of the first variants of COVID-19 have been heterogeneous across countries, regions, and social classes of the affected population.
• Monitoring trends over a two-year pandemic period is essential to understanding how to improve the local response to a massive health threat.
• Identifying the characteristics of affected populations, such as those with co-morbidities and social deprivation, is necessary to define targeted public health interventions.
• Overcoming barriers to care for migrants from countries with high migration pressure, especially those who have recently arrived, could reduce severe outcomes, and improve equity in healthcare.

## Background

The COVID-19 pandemic has had a disproportionate impact on the global burden of disease, particularly on vulnerable populations within and between countries, and has exacerbated health and social inequalities worldwide [1]. Comorbidities, such as chronic kidney disease, diabetes, cardiovascular disease, and chronic respiratory disease, have been shown to increase the risk of hospitalization, intensive care unit (ICU) admission, and death. At the onset of the pandemic, it was estimated that 1.7 billion (uncertainty interval from 1.0 to 2.4) people worldwide had at least one underlying clinical condition that put them at an increased risk of in-hospital severe acute COVID-19 [2–4]. Immigrants and ethnic minorities, who have a higher prevalence of some chronic conditions such as diabetes and obesity [5, 6], may experience a worse clinical course of COVID-19 [7–10]. Moreover, social determinants of health such as social deprivation, low income, and racial discrimination play a relevant role in this association because of their documented impact on self-care management of major chronic conditions and on access to primary healthcare and preventive services [11, 12]. In both the USA and the WHO European Region, people of foreign origin and ethnic minorities were found to have worse outcomes than the native population, even after adjustment for clinical conditions and despite differences in the categorization of the study population, with ethnic minorities being studied in the USA and United Kingdom and migrants being studied in Europe [13, 14]. In addition, patterns of risk of infection and severe outcomes varied over time during the different phases of the pandemic, both in the general population and in vulnerable groups [15, 16]. An important factor was the introduction of vaccination as a public health policy, which was successful in preventing severe outcomes [17]. In Italy the vaccination campaign was launched in January 2021, focusing on high-risk groups (healthcare workers, the elderly, people with comorbidities) and have been gradually extended to the general population Unfortunately, several factors such as the lack of legal entitlement to healthcare or administrative barriers, ineffective inclusion in vaccination campaigns, vaccine safety concerns, vaccine hesitancy and mistrust of public health authorities among certain social and ethnic groups, lead to a suboptimal vaccination coverage among vulnerable groups, compared with the general population [18–20].

The present study was conducted in Emilia-Romagna (E-R) region, the sixth most populous region of Italy and one of the first and most affected by the pandemic in Europe, within the context of the EU’s Horizon 2020 research project called ORCHESTRA (Connecting European Cohorts to increase common and effective response to SARS-CoV-2 pandemic www.orchestra-cohort.eu). The study aimed to investigate the risk of hospitalization and the associated short-term outcomes (severe disease and all-cause mortality) during the acute phase of COVID-19 (30 days after SARS-CoV-2 infection) over the first two years of the pandemic.

The study assessed the impact of social determinants of health, such as deprivation and citizenship, in addition to clinical characteristics and COVID vaccination status.

## Methods

### Data sources

Data sources were extracted from the E-R healthcare administrative databases. These include the regional COVID-19 notification system, the hospital discharge records, the Regional Population Health Register (RPHR), integrated with the Regional Health Insurance Card System (RHICS) [21] for the address of residence and information on deaths. The 2011 General Census of Population and Housing was used for the variables constituting the Deprivation Index (DI).

Data sources were linked using a pseudonymized numerical key that uniquely identifies all individuals within the RPHR and the regional health administrative databases, including RHICS and COVID-19 notifications. The DI was linked using census block numbers corresponding to the study subjects’ place of residence.

### Study design

This is an observational retrospective population-based cohort study. The study included all consecutive subjects who tested positive for SARS‑CoV‑2 in E-R for the first time (molecular or antigen test) in the period from 1st February 2020 to 28th February 2022. To be eligible for inclusion, subjects had to be at least 18 years old at the time of diagnosis and had to have resided continuously in E-R for 365 days prior to diagnosis.

### Outcomes

During the acute phase of the disease (0–30 days after SARS-CoV-2 infection diagnosis), the study outcomes observed were hospitalization, occurrence of in-hospital severe acute COVID-19, and all-cause mortality. The hospitalization outcome included all acute care hospitalizations for any cause. In-hospital severe acute COVID-19 was assigned algorithmically based on diagnoses and procedures reported in-hospital discharge records (Supplementary Fig. 1 and Supplementary Table 1 in Additional File 1). All-cause mortality included both in-hospital and out-of-hospital mortality. All outcomes were dichotomous.

### Explanatory variables

The study considered sex and age group (18–39, 40–49, 50–59, 60–69, 70–79, ≥ 80) as potential individual risk factors of interest. In addition, the time period was divided into four categories based on the prevalent circulating SARS-CoV-2 variants: the ‘pre-Alpha’ period from February 2020 to January 2021, the ‘Alpha’ period from February to June 2021, the ‘Delta’ period from July to December 2021, and the ‘Omicron’ period from January to February 2022. SARS-CoV-2 variants were associated with time periods by determining the prevalent circulating variant (a variant that accounts for more than 50% of SARS-CoV-2 infections) in each calendar month through official genetic sequencing surveys conducted by the Italian Istituto Superiore di Sanità [22]. The study also considered ecological and socioeconomic variables such as the DI, a summary measure of social and material deprivation expressed in 5 quintiles of the population, and immigration status defined as foreigners coming from high migratory pressure countries (HMPCs) or Italians and foreigners from low migratory pressure countries (LMPCs). Additionally, the duration of residence in E-R was calculated as a proxy for social integration.

A risk of hospitalization or death score (RHDS) based on comorbidities and previous access to healthcare resources was also included in the analysis. Vaccination status was defined as follows: recently vaccinated, if subjects received at least one dose of vaccine and tested positive between 14 and 120 days after the last injection; not recently vaccinated, if subjects received at least one dose more than 120 days before infection; not vaccinated, if subjects did not receive any dose of vaccine before infection or received the first dose of vaccine within 14 days before infection. Supplementary Table 2 in Additional File 1 provides a detailed description of all explanatory variables, their classification, and related sources.

Finally, we decided to stratify by sex throughout the analysis, because in previous studies sex emerged as an influential characteristic on most severe Covid-19 outcomes [23] and also showed signs of being an effect modifier of other influential characteristics [24]. In addition, the preliminary assessment of the data revealed heterogeneity that needs to be further investigated. Finally, a more in-depth analysis of the effects by sex stratification allowed a more precise description of the outcomes and their determinants.

### Data analysis

Descriptive statistics, including mean, median, interquartile range (IQR) and frequency distributions, were used to assess the characteristics of the cohort. Multivariable regression models were performed to assess the association between the three outcomes and the following independent variables: age, citizenship, DI, RHDS, and vaccination status. Results were expressed as risk ratios (RR), and uncertainty was reported as the 95% confidence interval (CI). A Poisson model was used for the analyses, with standard errors corrected using the robust sandwich method. All analyses were stratified by sex to allow a more accurate description of outcomes and their determinants. In a supplementary analysis we also stratified by time period. In the main analysis, citizenship was considered as a dichotomous variable: high or low migratory pressure country of origin. In a supplementary analysis, citizenship was considered in relation to duration of residence in E-R, as a proxy for integration and capacity to navigate the regional health system, particularly in relation to primary healthcare services. No stratification for pandemic period has been performed in this last supplementary analysis. Another supplementary analysis was performed using more disaggregated data on citizenship, as areas of origin were divided into 7 groups (Central Eastern Europe, North Africa, Sub-Saharan Africa, South Central America, Central Western Asia, Eastern Asia). Finally, the listwise deletion method was used to deal with missing data.

## Results

### Descriptive analysis

Between February 2020 and February 2022, 859,653 people tested positive for SARS-CoV-2 in E-R and were included in this cohort study. Among them, 47.8% were males and 52.2% females; 9.6% of them were citizen from HMPCs while immigrants coming from LMPCs accounted for the 0.3%, representing 2.7% of all cases with non-Italian citizenship. Duplicate or incomplete records accounted for about 0.01% of the total.

**Fig. 1:**
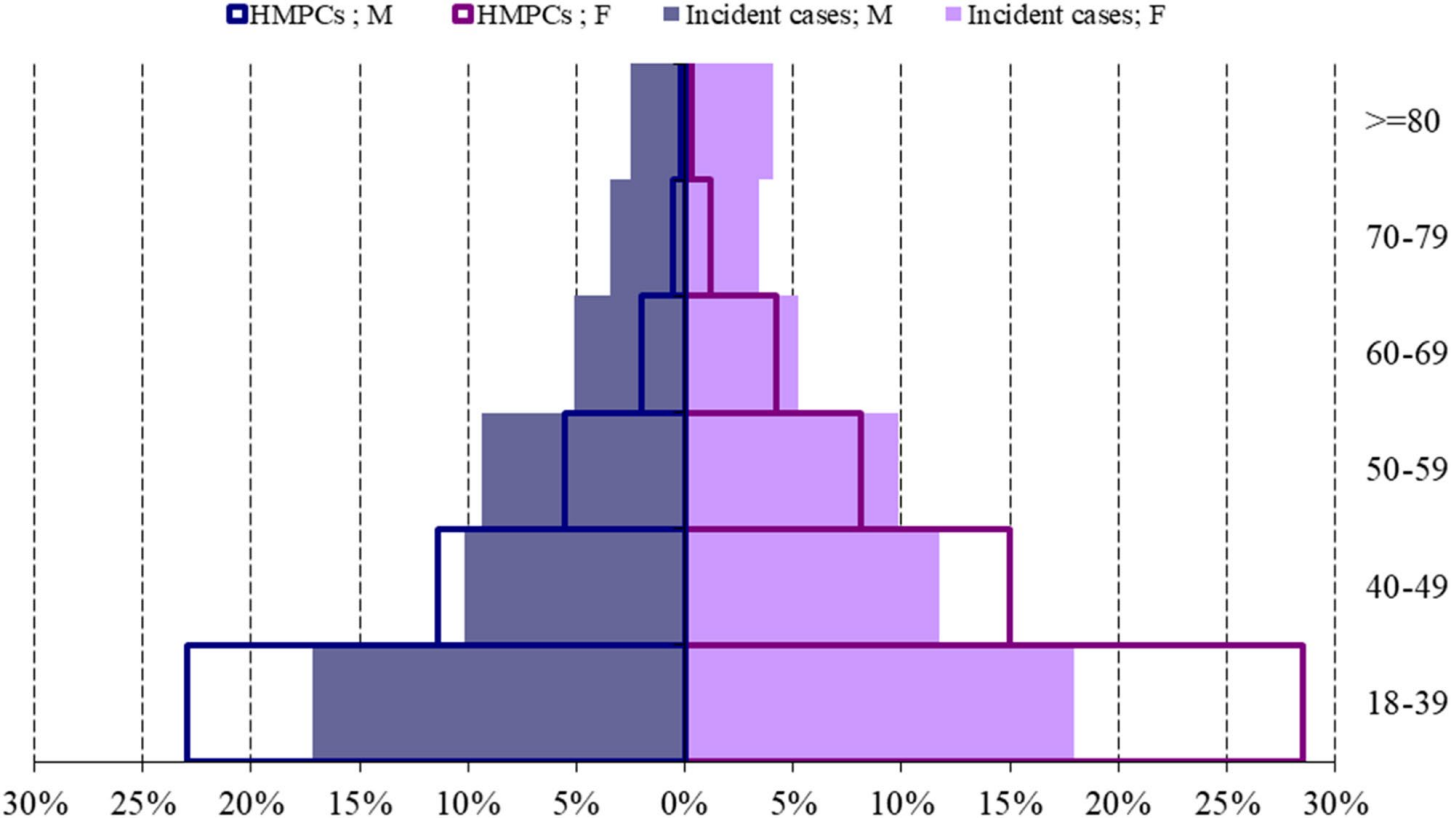
Age pyramid of the incident COVID-19 cases by citizenship (% on total population and immigrants), sex, and age group. Emilia-Romagna region, February 2020-February 2022. *Notes*: HMPCs = high migratory pressure countries; M = males; F = females. The age pyramid is expressed as a percentage of the total number of incident cases (*n* = 859,653) and of the total number of incident cases within HMPCs (*n* = 82648). (base = age group 18–39 years, superscript = age group > = 80 years)

The age group composition of the total incident cases is shown in Fig. 1; Table 1.

**Table 1:** Sociodemographic characteristics of SARS-CoV-2 incident cases: absolute numbers with column percentages of differences by sex. Emilia-Romagna region, February 2020-February 2022

All incident cases			
	Males (*n* = 410,553)	Females (*n* = 449,100)	All subjects (*n* = 859,653)
**Age**	**Males**	**Females**	Total
Median (IQR)	46 (33–58)	47 (34–59)	47 (34–59)
Mean (SD)	47 (17.8)	48.3 (18.5)	47.7 (18.2)
Min - Max	18–107	18–108	18–108
**Age group**	**Males**	**Females**	**Total**
18–39	147,72 (35.9%)	154,372 (34.4%)	301,944 (35.1%)
40–49	87,529 (21.3%)	100,824 (22.5%)	188,353 (21.9%)
50–59	80,597 (19.6%)	84,546 (18.8%)	165,143 (19.2%)
60–69	43,866 (10.7%)	44,702 (10.0%)	88,568 (10.3%)
70–79	29,369 (7.2%)	29,695 (6.6%)	59,064 (6.9%)
>=80	21,620 (5.3%)	34,961 (7.8%)	56,581 (6.6%)
**Risk of hospitalization or death score**	**Males**	**Females**	**Total**
Low	335,447 (81.7%)	379,733 (84.6%)	715,18 (83.2%)
Moderate	40,633 (9.9%)	33,751 (7.5%)	74,384 (8.7%)
High	11,355 (2.8%)	11,976 (2.7%)	23,331 (2.7%)
Very high	12,240 (3.0%)	14,689 (3.3%)	26,929 (3.1%)
Missing	10,878 (2.7%)	8,951 (2.0%)	19,829 (2.3%)
**Vaccination status at diagnosis**	**Males**	**Females**	**Total**
Not vaccinated	220,676 (53.7%)	236,312 (52.6%)	456,988 (53.2%)
Not recently vaccinated	102,402 (24.9%)	105,812 (23.6%)	208,214 (24.2%)
Recently vaccinated	87,475 (21.3%)	106,976 (23.8%)	194,451 (22.6%)
**Deprivation Index - population quintiles**	**Males**	**Females**	**Total**
1 - Least deprived quintile	83,807 (20.4%)	89,470 (19.9%)	173,277 (20.2%)
2	78,547 (19.1%)	85,389 (19.0%)	163,936 (19.1%)
3	79,354 (19.3%)	86,736 (19.3%)	166,090 (19.3%)
4	77,389 (18.8%)	85,653 (19.1%)	163,042 (19.0%)
5 – Most deprived quintile	77,793 (19.0%)	87,023 (19.4%)	164,816 (19.2%)
Missing	13,663 (3.3%)	14,829 (3.3%)	28,492 (3.3%)
**Immigration status**	**Males**	**Females**	**Total**
Italians	374,474 (91.2%)	400,233 (89.1%)	774,707 (90.1%)
other LMPCs residents	845(0.2)	1,508 (0.3)	2,353 (0.3)
HMPCs resident from 1 to 5 years	7.893 (1.9%)	9,556 (2.1%)	17,449 (2.0%)
HMPCs resident from 6 to 8 years	10,261 (2.5%)	14,506 (3.2%)	24,767 (2.9%)
HMPCs resident from more than 8 years	17,030 (4.1%)	23,213 (5.2%)	40,243 (4.7%)
Missing	50 (0.0%)	84 (0.0%)	134 (0.0%)
**Years of residency of HMPCs**	**Males**	**Females**	**Total**
Median (IQR)	7.9 (5.3–15.4)	8.0 (5.5–14.4)	8.0 (5.4–14.7)
Mean (SD)	14.3 (15.1)	13.9 (14.8)	14.1 (14.9)

Notes: HMPCs = high migratory pressure countries; LMPCs = low migratory pressure countries; SD = standard deviation; IQR = interquartile range

Most subjects were less than 50 years old at diagnosis, for both sexes (57.2% for males and 56.9% for females). Among the higher age groups (70 and over) females were more numerous than males, both for Italians and foreigners. Among the residents from HMPCs, the 51.5% of the immigrant population belong to the 18–39 age group, compared with the 38.9% of Italians.

Table 1 shows that most subjects were at low risk of hospitalization, with 81.7% of males and 84.6% of females falling into this category. Furthermore, 53.2% of the subjects were not vaccinated at the time of infection, while 24.2% were not recently vaccinated and 22.6% were recently vaccinated.

The median duration of residency among residents with citizenship from HMPCs was 8.0 years (IQR from 5.4 to 14.7), and about 65% of them had resided in E-R for more than 5 years.

For 134 immigrants, it was not possible to calculate the time of residency, as the date of beginning of residence was not available in the administrative databases. Of these people, 62.7% were males and 37.3% were females. The majority (51.5%) came from Central Eastern Europe, followed by 21.5% from Sub-Saharan Africa and 15.6% from Central Western Asia.

Regarding the area of origin of immigrants from HMPCs, 55.9% were from Central Eastern Europe, followed by 14.6% from North Africa and 9.8% from Central Western Asia. It is worth noting that Sub-Saharan Africans were more likely to have immigrated in the last five years (38.6%) than other groups (Supplementary Table 3 in Additional File 1). As presented in Table 2, incident cases in the pre-Alpha period were on average older than cases in other pandemic periods, with a higher mean age among females in all periods and a general downward age gradient over the two pandemic years.

**Table 2:** Number of incident cases of SARS-CoV-2 per period (absolute and row %) and mean age, by sex. Emilia-Romagna region, February 2020-February 2022

	Males			Females			Total
Period	*N*	Mean Age	Row %	*N*	Mean Age	Row %	*N*
pre-Alpha	**85**,**888**	51,7	47.4	**95**,**234**	54.2	52.6	**181**,**122**
Alpha	**62**,**859**	48.5	49.7	**63**,**728**	49.9	50.3	**126**,**587**
Delta	**69**,**463**	45.0	49.1	**71**,**881**	46.2	50.9	**141**,**344**
Omicron	**192**,**343**	45.2	46.8	**218**,**257**	45.9	53.2	**410**,**600**
Total	**410**,**553**			**449**,**100**			**859**,**653**

Notes: pre-Alpha’ period from February 2020 to January 2021; ‘Alpha’ period from February to June 2021; ‘Delta’ period from July to December 2021; ‘Omicron’ period from January to February 2022

In both males and females, most cases occurred during the period of spread of the Omicron variant (January - February 2022).

**Fig. 2:**
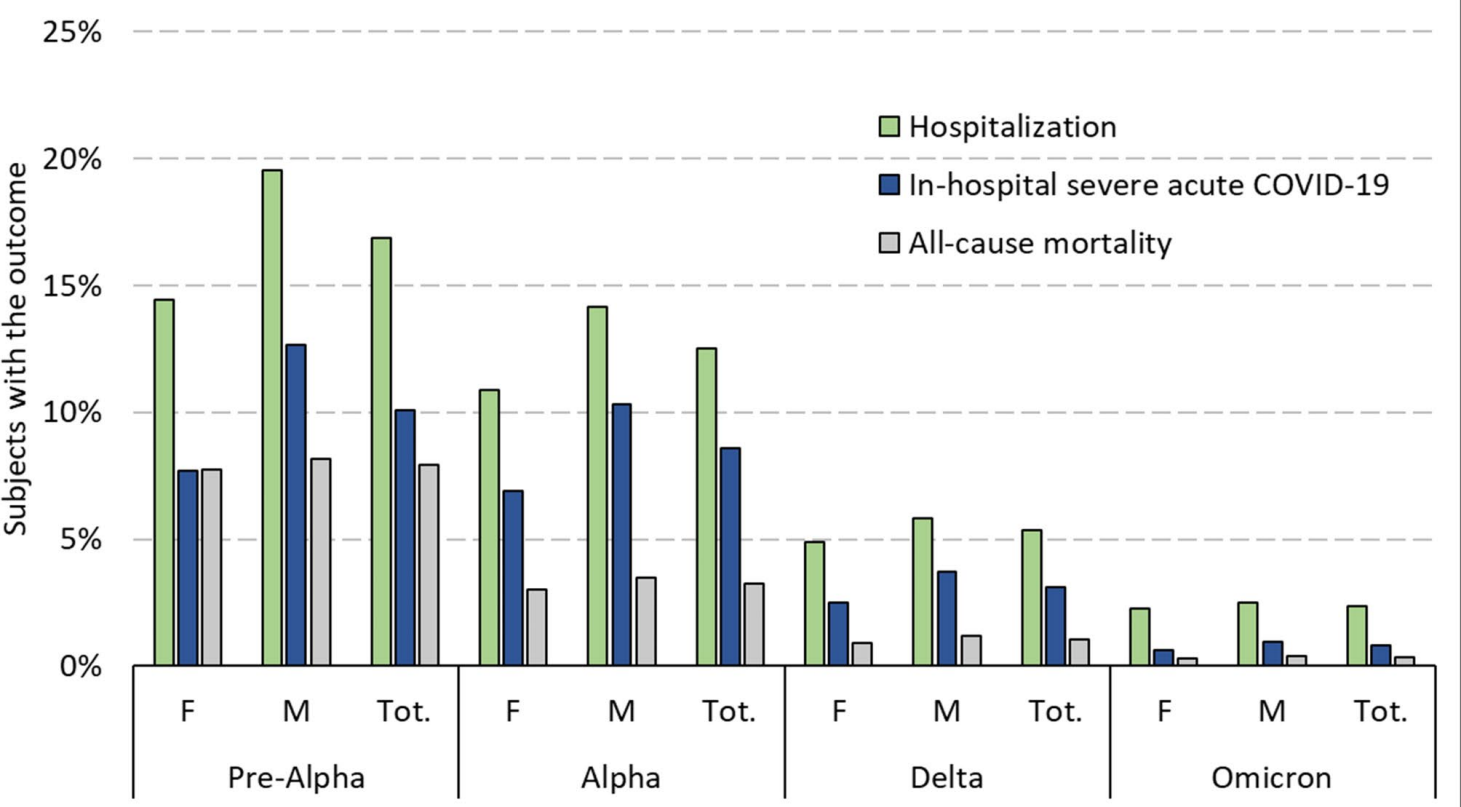
Frequencies of outcomes (%) among SARS-CoV-2 incident cases per period. Emilia-Romagna region, February 2020-February 2022. Notes: M = males; F = females

Figure 2 shows how the proportion of subjects developing any of the acute outcomes among incident cases decreased significantly over the two years.

Mortality decreased from 8.2% for males and 7.8% for females in the pre-Alpha period to 0.4% and 0.3%, respectively, in the Omicron period. The sex differences in each outcome, especially hospitalization and in-hospital severe disease, also decreased over time.

### Model results

The main analysis was performed to estimate the risk ratios for hospitalization, in-hospital disease severity and all-cause mortality, among residents living in E-R region who tested positive for SARS-Cov-2 between February 2020 and February 2022.

Tables 3, 4 and 5 show the results of the analysis for hospitalization, in-hospital disease severity, and all-cause mortality, during the entire pandemic period.

**Table 3:** Association between explanatory variables and risk of hospitalization, by sex. Emilia-Romagna region, February 2020-February 2022

HOSPITALIZATION							
		**Males RR** **[n. of events = 34**,**556]**	**95% CI**	p-value	**Females RR** **[n. of events = 29**,**175]**	**95% CI**	p-value
Age groups	**18–39**	1.00			1.00		
	**40–49**	**2.91**	**2.74–3.09**	**	**1.16**	**1.09–1.23**	**
	**50–59**	**5.68**	**5.38-6.00**	**	**2.21**	**2.10–2.33**	**
	**60–69**	**10.74**	**10.16–11.35**	**	**4.71**	**4.48–4.95**	**
	**70–79**	**14.66**	**13.82–15.56**	**	**7.57**	**7.18–7.99**	**
	**>=80**	**16.16**	**15.20-17.18**	**	**8.10**	**7.64–8.58**	**
Citizenship	**Italians and LMPCs**	1.00			1.00		
	**HMPCs**	**1.55**	**1.48–1.62**	**	**1.82**	**1.75–1.89**	**
Deprivation index - population quintiles	**1- Least deprived**	1.00			1.00		
	**2**	1.01	0.98–1.04		1.02	0.99–1.06	
	**3**	1.00	0.98–1.03		1.03	0.99–1.06	
	**4**	**1.04**	**1.01–1.07**	*	1.01	0.97–1.04	
	**5- Most deprived**	**1.04**	**1.01–1.07**	**	1.03	1.00-1.06	
	**Missing**	1.05	0.99–1.10		0.98	0.92–1.04	
Risk of hospitalization or death score	**Low**	1.00			1.00		
	**Moderate**	**1.86**	**1.80–1.92**	**	**2.25**	**2.17–2.34**	**
	**High**	**2.73**	**2.63–2.84**	**	**3.05**	**2.91–3.20**	**
	**Very high**	**3.27**	**3.15–3.39**	**	**3.37**	**3.21–3.52**	**
	**Missing**	**1.32**	**1.23–1.43**	**	**1.18**	**1.08–1.29**	**
Vaccination status	**Not vaccinated**	1.00			1.00		
	**Not recently vaccinated**	**0.30**	0.29–0.31	**	**0.33**	**0.32–0.35**	**
	**Recently vaccinated**	**0.26**	0.25–0.27	**	**0.32**	**0.31–0.33**	**

Notes: ** *p* < 0.01; * *p* < 0.05; RR = risk ratio; CI = confidence interval; HMPCs = high migratory pressure countries; LMPCs = low migratory pressure countries

**Table 4:** Associations between explanatory variables and risk of in-hospital severe disease, by sex. Emilia-Romagna region, February 2020-February 2022

IN-HOSPITAL SEVERITY							
		**Males RR** **[n. of events = 21**,**834]**	95% CI	p-value	**Females RR** **[n. of events = 14**,**937]**	95% CI	p-value
Age groups	**18–39**	1.00			1.00		
	**40–49**	**4.67**	**4.25–5.13**	**	**3.80**	**3.33–4.33**	**
	**50–59**	**9.74**	**8.93–10.63**	**	**9.13**	**8.09–10.30**	**
	**60–69**	**20.42**	**18.72–22.28**	**	**21.54**	**19.13–24.26**	**
	**70–79**	**29.63**	**27.05–32.45**	**	**34.76**	**30.76–39.27**	**
	**>=80**	**32.55**	**29.62–35.77**	**	**33.64**	**29.61–38.22**	**
Citizenship	**Italians and LMPCs**	1.00			1.00		
	**HMPCs**	**1.33**	**1.26–1.42**	**	**1.65**	**1.55–1.75**	**
Deprivation index – population quintiles	**1- Least deprived**	1.00			1.00		
	**2**	1.02	0.98–1.06		1.02	0.97–1.07	
	**3**	1.01	0.97–1.05		1.01	0.97–1.06	
	**4**	1.03	0.99–1.07		1.01	0.96–1.06	
	**5- Most deprived**	1.04	1.00-1.08		**1.06**	**1.01–1.11**	*
	**Missing**	1.01	0.94–1.08		**0.88**	**0.80–0.96**	**
Risk of hospitalization or death score	**Low**	1.00			1.00		
	**Moderate**	**1.60**	**1.54–1.67**	**	**2.08**	**1.97–2.19**	**
	**High**	**2.17**	**2.06–2.28**	**	**2.75**	**2.58–2.94**	**
	**Very high**	**2.54**	**2.41–2.67**	**	**3.14**	**2.94–3.35**	**
	**Missing**	**1.18**	**1.06–1.30**	**	1.12	0.98–1.28	
Vaccination status	**Not vaccinated**	1.00			1.00		
	**Not recently vaccinated**	**0.22**	**0.20–0.23**	**	**0.23**	**0.21–0.24**	**
	**Recently vaccinated**	**0.14**	**0.13–0.15**	**	**0.16**	**0.15–0.17**	**

Notes: ** *p* < 0.01; * *p* < 0.05; RR = risk ratio; CI = confidence interval; HMPCs = high migratory pressure countries; LMPCs = low migratory pressure countries

**Table 5:** Associations between explanatory variables and risk of all-cause mortality, by sex. Emilia-Romagna region, February 2020-February 2022

ALL-CAUSE MORTALITY							
		**Males RR** **[n. of events = 10**,**807]**	**IC95%**	p-value	**Females RR** **[n. of events = 10.659]**	**IC95%**	p-value
Age groups	**18–39**	1.00			1.00		
	**40–49**	**4.68**	**2.82–7.78**	**	**3.73**	**1.79–7.76**	**
	**50–59**	**18.53**	**11.74–29.25**	**	**17.68**	**9.26–33.77**	**
	**60–69**	**70.57**	**44.94-110.81**	**	**66.59**	**35.32-125.55**	**
	**70–79**	**163.44**	**103.55-257.98**	**	**163.22**	**86.23-308.95**	**
	**>=80**	**277.22**	**175.31-438.37**	**	**285.34**	**150.36-541.52**	**
Citizenship	**Italians and LMPCs**	1.00			1.00		
	**HMPC**	0.85	0.71–1.02		**0.73**	**0.59–0.90**	**
Deprivation index – population quintiles	**1- Least deprived**	1.00			1.00		
	**2**	**0.94**	**0.88-1**	*	1.00	0.93–1.07	
	**3**	0.94	0.88-1		0.94	0.88–1.02	
	**4**	1.00	0.94–1.06		0.98	0.91–1.05	
	**5- Most deprived**	1.00	0.94–1.06		1.06	0.99–1.14	
	**Missing**	0.95	0.85–1.07		0.89	0.78–1.01	
Risk of hospitalization or death score	**Low**	1.00			1.00		
	**Moderate**	**2.95**	**2.64–3.30**	**	**4.06**	**3.52–4.67**	**
	**High**	**5.97**	**5.30–6.72**	**	**8.17**	**7.03–9.49**	**
	**Very high**	**8.72**	**7.76–9.79**	**	**12.08**	**10.42–14.01**	**
	**Missing**	**3.84**	**3.17–4.66**	**	**6.5**	**5.37–7.88**	**
Vaccination status	**Not vaccinated**	1.00			1.00		
	**Not recently vaccinated**	**0.34**	**0.32–0.37**	**	**0.37**	**0.33–0.40**	**
	**Recently vaccinated**	**0.15**	**0.14–0.17**	**	**0.19**	**0.18–0.21**	**

Notes: ** *p* < 0.01; * *p* < 0.05; RR = risk ratio; CI = confidence interval; HMPCs = high migratory pressure countries; LMPCs = low migratory pressure countries

Results by time periods are shown in Supplementary Tables 4, 5 and 6 (Additional File 1).

The risk of being hospitalized increased with increasing age, in particular doubling from the age group 50–59 years to 60–69 years (from RR = 5.68 95% CI = 5.38-6.00 to RR = 10.74 95% CI = 10.16–11.35 for males and from RR = 2.21 95% CI = 2.10–2.33 to RR = 4.71 95% CI = 4.48–4.95 for females).

Moreover, the risk ratio for hospitalization in older age groups (> 80 years) was twice as high in males (RR = 16.2 95% CI = 15.2–17.2) than in females (RR = 8.1, 95% CI = 7.6–8.6), especially in the pre-Alpha, Delta and Omicron periods (Supplementary Table 4 in Additional File 1).

The risk ratio of in-hospital severe disease also increased with increasing age especially after 59 years (RR = 20.42 95% CI = 18.72–22.28 among males and RR = 21.54 95% CI = 19.13–24.26 among females) with an indication of a higher RR in older females compared to older males (RR = 34.76 95% CI = 30.76–39.27 among 70–79-year-old females, RR = 29.63 95% CI = 27.05–32.45 among 70–79 year old males).

Concerning all-cause mortality, the RRs increased constantly with age, especially after 70 years both in males (from RR = 70.57 95% CI = 44.94-110.81 among 60–69 to 163.44 95% CI = 103.55-257.98 among 70–79) and in females (from RR = 66.59 95% CI = 35.32-125.55 among 60–69 to RR = 163.22 95% CI = 86.23-308.95 among 70–79) with higher risk ratios in males compared to females especially during the first pandemic period (Supplementary Table 6 in Additional File 1).

An increasing gradient of hospitalization risk was observed in both males and females with moderate (RR = 1.86 95% CI = 1.80–1.92 for males, RR = 2.25 95% CI = 2.17–2.34 for females), high (RR = 2.73 95% CI = 2.63–2.84 for males, RR = 3.05 95% CI = 2.91–3.20 for females) or very high RHDS (RR = 3.27 95% CI = 3.15–3.39 for males, RR = 3.37 95% CI = 3.21–3.52 for females); the same trend is observed for disease severity and even for all-cause mortality (RR of very high RHDS = 8.72 95% CI = 7.76–9.79 for males, RR of very high RHDS = 12.08 95% CI = 10.42–14.01 for females) during all pandemic periods, with higher point estimates for females.

In relation to vaccination status (from none to recent) a noticeably lower risk ratio of hospitalization, serious illness and mortality was observed in those vaccinated with at least one dose between 14 and 120 days before the infection.

Compared to never-vaccinated, recently vaccinated males were 74% more protected against the risk of hospitalization while females were 68% more protected.

The protection of recent vaccination increased against in-hospital severe outcomes (RR = 0.14 95% CI = 0.13–0.15 among males, RR = 0.16 95% CI = 0.15–0.17 among females); in relation to mortality, the protective effect of vaccination decreased especially if vaccination was not recent and particularly in the Omicron period (RR = 0.64 95% CI = 0.52–0.78 in males and RR = 0.65 95% CI = 0.54–0.78 in females; data shown in Supplementary Table 4 in Additional File 1).

The risk of hospitalization and disease severity was higher for residents from HMPCs than for Italians and other residents from LMPCs in almost all periods with higher RR values for foreigners females (RR = 1.82 95% CI = 1.75–1.89) compared to males (RR = 1.55 95% CI = 1.48–1.62), as well as the risk of in-hospital severe disease (RR for females 1.65 95% CI = 1.55–1.75 compared to males RR = 1.33 CI 95% 1.26–1.42); less clear evidence was observed only for the pre-Alpha wave (RR for males 1.08 95% CI = 0.98–1.18) and the Omicron wave in both sexes (RR = 1.26 95% CI = 0.97–1.64 for males and RR = 1.21 95% CI = 0.93–1.56 for females). (Supplementary Table 5 in Additional File 1).

Considering the duration of residence in the E-R region, the risk ratio for in-hospital severe outcomes in HMPCs seems to be more pronounced among those who arrived less than 5 years ago in both sexes concerning hospitalization (RR = 1.92, 95% CI = 1.76-2.00 for males, and RR = 2.40, 95% CI = 2.23–2.59 for females) and, concerning in-hospital severe disease, in females (RR = 1.81 95% CI = 1.59–2.07) (Supplementary Table 7 in Additional File 1).

Being a resident from HMPCs had a general protective effect on all-cause mortality, especially among females (RR = 0.73 95% CI = 0.59–0.9), while male residents of HMPCs had a 66% increased risk of all-cause mortality in the period July-December 2021 compared with LMPCs.

Immigrant residents from less than five years showed a lower risk ratio of all-cause mortality both among males (RR = 0.61 95% CI = 0.38–0.99) and females (RR = 0.51 95% CI = 0.29–0.89) while in relation to area of origin, only males from Sub-Saharan Africa showed an 82% increased risk compared to residents from LMPCs (Supplementary Table 5 in Additional File 1).

In relation to the Deprivation Index, occasionally the most deprived classes had an indication of higher risk ratio of hospitalization (RR for males = 1.04 95% CI = 1.01–1.07) or in-hospital disease severity (RR for females = 1.06 95% CI = 1.01–1.11) during the entire pandemic period.

## Discussion

### Main results

The results of this study, which used data collected over two years and related to the different phases of the pandemic, provide an overall view of the progression of acute COVID-19 outcomes. The intensity of the effects was particularly high in the early phases and decreased significantly over the two-year period. During the pandemic period, the risk of in-hospital severe outcomes in the acute phase of COVID-19 increased with age, particularly among males. The level of RHDS predicted worse outcomes in both sexes. Immigrants from HPMCs, especially females, showed a higher risk of hospitalization and in-hospital severe disease, while the risk of all-cause mortality was lower compared with residents from LMPCs. However, this was not the case for immigrants from specific areas of origin and during certain pandemic periods. Other socioeconomic indicators, such as the DI, did not appear to have an impact on acute outcomes, at least not in the first 30 days after infection. Conversely, vaccination against COVID-19 was shown to have a strong protective effect against all outcomes of acute infection, supporting the importance of vaccination campaigns.

### Interpretation in the framework of current literature

Covering a two-year period the study allowed to highlight not only the observed trend in outcomes but also the risk associated with various clinical, demographic, and social characteristics analyzed jointly. As widely described in the current literature, the risk of hospitalization and in-hospital severe outcomes increases with age. All-cause mortality in elderly was much higher, consistently with their worse health conditions [25–27]. Our data also confirm that, regarding comorbidities, people with multiple chronic diseases and higher healthcare resources utilization (very high and high RHDS) were more at risk of in-hospital severe acute outcomes. This risk has already been assessed in previous research, for example on diabetes, obesity, and hypertension [28, 29]. Data also show a good performance of RHDS, like many other comorbidity indexes, as a reliable tool to identify population at higher risk of experiencing at least one in-hospital severe COVID-19 outcome [23, 30].

It is acknowledged that the occurrence of death and disease severity is generally higher in males with COVID-19 than in females [31] while the increasing care complexity (measured with the RHDS) seems to have a greater impact on severe outcomes among females than in males, in our data.

The significant decrease in hospitalizations, in-hospital severe diseases, and all-cause mortality over the two years of the pandemic has been documented in literature, although few data are available at national [32] and regional level [33–35]. This trend has been attributed to the implementation of COVID-19 vaccination campaigns [36], which began in Italy in January 2021, initially targeting high-risk groups and then extending to the general population. Furthermore, improvements in other public health preventive measures or in clinical case management may have contributed. This finding is particularly evident when looking at the trend from the Alpha phase to the Delta phase (i.e., from February-June 2021 to July-December 2021).

Being a citizen from HMPCs has been shown to increase the risk of hospitalization and COVID-19 in-hospital severe disease. This is confirmed by national data and has also been reported in countries with a similar immigration history [37, 38]. 

The increased risk of acute outcomes for people from HMPCs was found to be particularly relevant among recently resident immigrants (0–8 years) and for hospitalization and in-hospital severe disease outcomes. This may be due to multiple barriers to primary care and preventive services, which may lead to delayed treatment seeking and hospitalization once infected. In contrast, foreigners from HMPCs seem to be slightly protected from mortality risk, which is probably due to the well-known healthy migrant effect [39, 40]. Moreover, among residents from HMPCs, Asians and Sub-Saharan Africans showed a particularly high risk of serious outcomes in both sexes. Although the estimates may be less reliable due to the smaller numbers of individuals in these subgroups, these findings are broadly in line with current evidence [41]. In contrast with similar studies conducted in Italy using the same socioeconomic indicator [42] the Deprivation Index did not provide significant evidence of increased risk of short-term outcomes, except for a small indication of an association between higher DI quintiles and in-hospital severe disease and all-cause mortality, particularly among females, and the two higher DI quintiles with hospitalization among males. Probably the social disadvantage could be considered less intense and harmful in the E-R compared to most Italian regions [43].

### Strengths, limitations, and perspectives

This study is part of the body of evidence on COVID-19 acute and post-acute outcomes and their determinants developed within the ORCHESTRA research project [44–46]. In particular, the present study provides important information about acute outcomes during the first two years of the pandemic period, when COVID-19 was considered a public health emergency by national and international authorities. To our knowledge, this is one of the first Italian studies on the acute outcome of COVID-19 in relation to clinical complexity, sociodemographic variables, citizenship, and deprivation. There are important limitations that need to be considered. First, the data on infections suffered from a notification deficit, especially in the first period, leading to an overestimation of lethality. More in general, the study is based on routinely collected health and sociodemographic data from health databases and official statistics, not for research purposes. This facilitated the availability of data but introduced a risk of low or inaccurate data quality. Moreover, the use of DI, which is updated to 2011 and based on specific dimensions aggregated to census blocks, may result in a poor capacity to represent the actual characteristics of social deprivation, and may introduce a risk of ecological bias. Other variables may also be affected by operationalization defects which may influence the estimates of association measures. Furthermore, another limitation is that the effect of specific comorbidities was not assessed.

## Conclusions

The present study describes the history of the national health emergency period due to COVID-19 pandemic, by examining health administrative databases from a region in northern Italy, to assess acute health outcomes and their determinants. The results highlight arising challenges and achievements from a public health perspective during the two-year pandemic period. The marked reduction in hospitalizations, in-hospital severe disease and all-cause mortality can be attributed in part to national and regional health policies, increased public awareness, mass vaccination campaigns and other non-pharmacological interventions.

However, as happened in many European countries, the COVID-19 pandemic disproportionately affected vulnerable populations, even in a universalistic and equity-oriented regional healthcare system [47] such as the E-R one. The present study allowed us to better identify disadvantaged groups and their clinical and sociodemographic characteristics. The results can have implications for health and social policies regarding primary care, prevention strategies, and epidemic preparedness plans, suggesting the implementation of targeted, gender-based and culturally competent interventions.

## Electronic supplementary material

Below is the link to the electronic supplementary material.

Supplementary Material 1

## Data Availability

Individual data supporting the findings of this study are not publicly available due to security measures to protect personal data of participants. Aggregated data and source code used for the analysis are available from the corresponding author upon reasonable request and with the written permission of the Emilia-Romagna Region.

## Abbreviations

CI: Confidence interval
IQR: Interquartile range
RR: Risk ratio
SD: Standard deviation
ICU: Intensive care unit
E-R: Emilia - Romagna
RPHR: Regional population health register
RHICS: Integrated with the regional health insurance card system
RHDS: Risk of hospitalization or death score
DI: Deprivation index
HMPCs: High migratory pressure countries
LMPCs: Low migratory pressure countries
