# Supplementary material for: Short-term acute outcomes by clinical and socioeconomic characteristics in adults with SARS-CoV-2: a population-based cohort study focused on the first two years of the COVID-19 pandemic
Source: Arch Public Health. 2025 Mar 24;83:76. doi: 10.1186/s13690-025-01537-z (PMC11931843; doi:10.1186/s13690-025-01537-z)
Supplement: Supplementary file 1 — Supplementary Material 1 [file 13690_2025_1537_MOESM1_ESM.docx]

**Supplementary Figure 1**. Algorithm for the identification of severe COVID-19 outcome


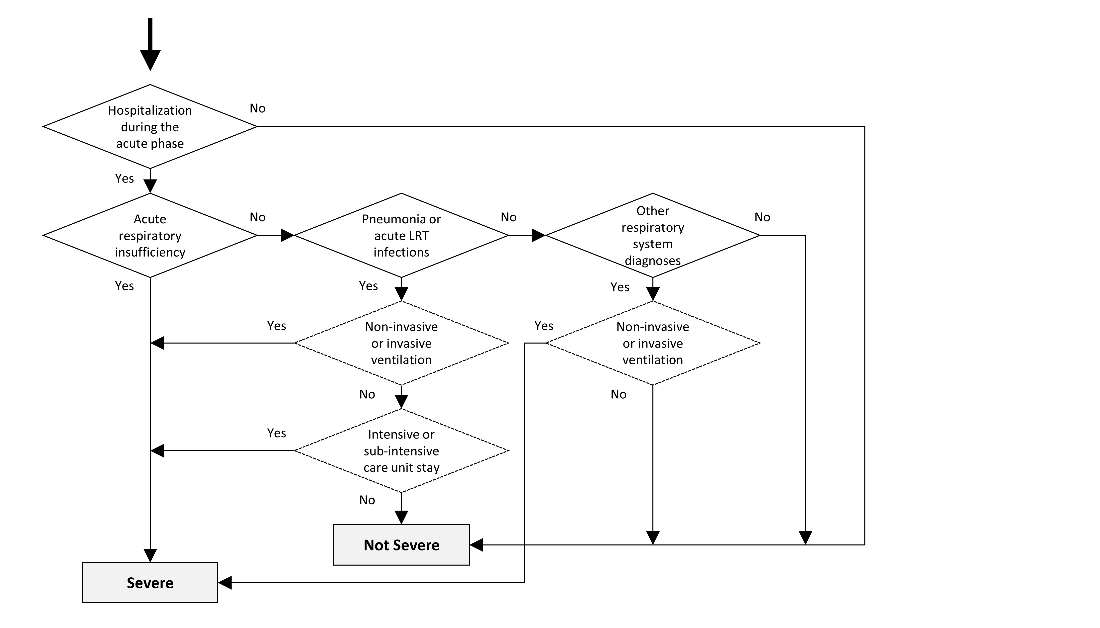


Notes: The decision tree shows the algorithm for the assignment of severe COVID-19 outcome. The rhombuses indicate binary decision rules based on hospitalizations, respiratory diagnoses (continuous border line) and ventilation procedures (dotted border line). The grey boxes indicate the assigned outcome. The data extraction criteria for each element in the decision tree are reported in Supplementary Table 1. LRT = lower respiratory tract.

**Supplementary Table 1**. Data extraction criteria for the identification of COVID-19 severity

| **Respiratory diagnoses and ventilation procedures** | **International Classification of Diseases (ICD-9-CM) codes / other criteria** |
| --- | --- |
| **Respiratory insufficiency** | ICD-9-CM principal and secondary diagnosis codes in hospital records: 518.81-82, 518.8, 518.9 (acute respiratory insufficiency) * |
| **Pneumonia or lower respiratory tract infections** | ICD-9-CM principal and secondary diagnosis codes in hospital records: 464.1-2, 466, 490, 491.22, 519.7 (acute tracheitis, bronchitis and bronchiolitis); 480-486, 487.0 (pneumonia); 487.1 (influenza with respiratory manifestations) * |
| **Other respiratory disease** | ICD-9-CM principal and secondary diagnosis codes in hospital records: 460-519, except 464.1-2, 466, 480-486, 487.0, 487.1, 490, 491.22, 518.81-82, 518.84, 518.9, 519.7 * |
| **Intensive care unit stay** | At least one day in intensive care units (national unit code 049) |
| **Sub-intensive care unit stay** | At least one day in sub-intensive care units (national unit code 094) |
| **Oxygen therapy** | ICD-9-CM principal and secondary diagnosis codes in hospital records: V46.2 OR  ICD-9-CM procedure codes in hospital records: 93.91, 93.96, 93.99 |
| **Non-invasive ventilation** | ICD-9-CM procedure codes in hospital records: 93.90 |
| **Invasive ventilation** | ICD-9-CM procedure codes in hospital records: 96.70, 96.71, 96.72 |

*Notes: ICD-9-CM = International Classification of Diseases, 9th revision - Clinical Modification, Versione Italiana 2007, which can be accessed at the following link:* [*https://www.salute.gov.it/imgs/C_17_pubblicazioni_2251_allegato.pdf*](https://www.salute.gov.it/imgs/C_17_pubblicazioni_2251_allegato.pdf) *(last accessed on 9th April 2024). * = Diagnosis codes 480.4, 518.9, and 519.7 were introduced by the Italian Ministry of Health in 2021.*

**Supplementary Table 2**. Description, classification, and source of variables.

| **Variable name** | **Description** | **Classification** | **Source** |
| --- | --- | --- | --- |
| Deprivation Index  (DI) | A summary measure of social and material deprivation based on five census variables (low level of education, unemployment, non-home ownership, single-parent family, and household overcrowding). | Classification in quintiles of the regional distribution. | (1) |
| Immigration status | Defined on the basis of the citizenship.  This categorization of exposure was chosen in order to better represent the socioeconomic demographic characteristics of these groups, that generally differ in relation to different migration context. | Classification of immigrants from :   - **low migratory pressure countries (LMPCs)** (Western Europe, North America, Oceania, Israel and Japan) and immigrants from - **high migratory pressure countries (HMPCs)** (Central-Eastern Europe, Central and South America, North Africa, sub-Saharan Africa, and Asia -except for Israel and Japan).     Classification of origin in 8 main areas will be performed by identifying these groups: Italy, other high developed countries (European western countries, Oceania, North America, Lichterstein, Swizerland, Andorre, Vatican), Central and Eastern Europe, Northern Africa, South Saharian Africa, Central Western Asia, East Asia. | (2-3) |
| Citizenship related to time of residency in Emilia-Romagna Region | Calculated with time in years from the first residency obtainment to the date of infection as a proxy of social integration and ability to navigate health care system of immigrant people from HMPCs and classified in relation to frequency of distribution. | Classification of immigrants in:   - HMPCs residents from 1 to 5 years - HMPCs residents from 6 to 8 years - HMPCs residents from more than 8 years. | Variable defined by the authors from administrative databases. |
| Risk of hospitalization or death score | In the E-R cohort, an individual risk of hospitalization or death score was also assigned using a previously developed standardized algorithm. This algorithm relies on a multivariable prediction model which estimates a punctual measure for the individual risk of hospitalization or death within the reference year. The risk of hospitalization and death is then scored according to a four-level scale: low, moderate, high, very high. This score is assigned yearly based on demographic and residence characteristics, comorbidities, and access to a wide spectrum of healthcare resources in a multiyear period before the reference year and is routinely available in E-R administrative databases. | Classification in four levels of risk of hospitalization or death:   - Low - Moderate - High - Very high. | (4) |
| Vaccination Status | Vaccination campaign in Italy, as in many European countries, started on 27th December 2020, giving priority to categories mainly at risk (elderly aged over 80 years, immunocompromised and patients with severe comorbidities, health workers), then giving vaccination access to other categories. In September 2021, vaccination has been offered to all people aged over 12 years old. | Classification of people in   - **Recently vaccinated** if they received at least one dose and tested positive between 14 and 120 days after the last injection. - **Not recently vaccinated** if they received at least one dose more than 120 days before infection. - **Not vaccinated** if they were not vaccinated before the infection or were vaccinated within 14 days before the infection. | Variable defined by the authors from administrative databases. |

*References:*

*1. Rosano A, Pacelli B, Zengarini N, Costa G, Cislaghi C, Caranci N. Update and review of the 2011 italian deprivation index calculated at the census section level. Epidemiol Prev. 2020;44(2–3):162–70.*

*2. Pacelli B, Zengarini N, Broccoli S, Caranci N, Spadea T, Di Girolamo C, et al. Differences in mortality by immigrant status in Italy. Results of the Italian Network of Longitudinal Metropolitan Studies. Eur J Epidemiol. 2016 Jul 26;31(7):691–701.*

*3. Etowa J, Hyman I, Dabone C, Mbagwu I, Ghose B, Sano Y, et al. Strengthening the Collection and Use of Disaggregated Data to Understand and Monitor the Risk and Burden of COVID-19 Among Racialized Populations. Can Stud Popul. 2021 Sep 4;48(2–3):201–16.*

*4. Louis DZ, Robeson M, McAna J, Maio V, Keith SW, Liu M, et al. Predicting risk of hospitalisation or death: a retrospective population-based analysis. BMJ Open. 2014 Sep 17;4(9):e005223.*

**Supplementary Table 3**. Distribution of SARS-CoV-2 cases in residents coming from HMPCs, by time of residency and area of origin. Emilia-Romagna region, February 2020 - February 2022.

|  | **Area of origin** | | | | | | | |
| --- | --- | --- | --- | --- | --- | --- | --- | --- |
|  | **Central Eastern Europe** | **North Africa** | **Sub Saharan Africa** | **South Central America** | **Central Western Asia** | **Eastern Asia** | **Total** | **Total** |
| **Time of residency** | % | % | % | % | % | % | % | **n** |
| **1-5** | 17.56 | 19.81 | 38.63 | 24.58 | 31.39 | 14.12 | 21.13 | **17,449** |
| **6-8** | 31.70 | 27.31 | 26.93 | 28.02 | 27.57 | 30.46 | 29.99 | **24,767** |
| **>8** | 50.59 | 52.81 | 34.00 | 47.38 | 40.78 | 55.30 | 48.72 | **40,243** |
| **Missing** | 0.15 | 0.07 | 0.44 | 0.02 | 0.26 | 0.12 | 0.16 | **134** |
| **Total %** | 100.00 | 100.00 | 100.00 | 100.00 | 100.00 | 100.00 | 100.00 | **82,593** |
| **Total n** | **46,210** | **12,096** | **6,576** | **4,793** | **8,081** | **4,837** | **82,593** |  |

**Supplementary Table 4**. Association between explanatory variables and risk of hospitalization, by sex and time period.Emilia-Romagna region, February 2020 - February 2022.

|  |  | **Pre-alpha period** | | **Alpha period** | |
| --- | --- | --- | --- | --- | --- |
|  |  | **Females**  **RR (95% CI)** | **Males**  **RR (95% CI)** | **Females**  **RR (95% CI)** | **Males**  **RR (95% CI)** |
| **Age groups** | 18-39 | 1.00 | 1.00 | 1.00 | 1.00 |
|  | 40-49 | 1.38 (1.25-1.52) | 3.18 (2.89-3.50) | 1.73 (1.53-1.97) | 3.51 (3.13-3.93) |
|  | 50-59 | 2.45 (2.24-2.67) | 5.78 (5.30-6.30) | 3.44 (3.07-3.84) | 6.51 (5.86-7.22) |
|  | 60-69 | 5.17 (4.75-5.61) | 10.03 (9.20-10.93) | 6.93 (6.23-7.70) | 10.89 (9.80-12.10) |
|  | 70-79 | 7.83 (7.17-8.54) | 13.17 (12.03-14.41) | 10.47 (9.38-11.7) | 14.76 (13.21-16.49) |
|  | ≥80 | 7.36 (6.71-8.07) | 13.56 (12.37-14.88) | 11.79 (10.46-13.28) | 16.50 (14.69-18.53) |
| **Citizenship** | LMPC and Italians | 1.00 | 1.00 | 1.00 | 1.00 |
|  | HMPC | 1.60 (1.50-1.71) | 1.34 (1.26-1.43) | 1.62 (1.5-1.75) | 1.38 (1.28-1.49) |
| **Deprivation index - population quintiles** | 1- Least deprived | 1.00 | 1.00 | 1.00 | 1.00 |
|  | 2 | 1.03 (0.99-1.08) | 1.03 (0.99-1.07) | 1.00 (0.94-1.07) | 0.99 (0.94-1.05) |
|  | 3 | 1.03 (0.99-1.08) | 0.98 (0.95-1.02) | 1.03 (0.96-1.10) | 1.02 (0.97-1.08) |
|  | 4 | 0.99 (0.94-1.03) | 1.03 (0.99-1.07) | 1.03 (0.97-1.10) | 1.03 (0.97-1.09) |
|  | 5- Most deprived | 0.98 (0.94-1.03) | 0.99 (0.96-1.03) | 1.13 (1.06-1.20) | 1.06 (1.00-1.12) |
|  | Missing | 0.86 (0.78-0.94) | 1.00 (0.93-1.08) | 1.13 (1.00-1.27) | 1.11 (1.00-1.22) |
| **Risk of hospitalization or death score** | Low | 1.00 | 1.00 | 1.00 | 1.00 |
|  | Moderate | 1.95 (1.85-2.06) | 1.60 (1.54-1.67) | 1.75 (1.63-1.87) | 1.48 (1.39-1.56) |
|  | High | 2.28 (2.14-2.43) | 2.05 (1.94-2.15) | 2.36 (2.17-2.57) | 2.03 (1.89-2.18) |
|  | Very high | 2.34 (2.20-2.50) | 2.22 (2.11-2.33) | 2.72 (2.50-2.95) | 2.39 (2.24-2.57) |
|  | Missing | 1.02 (0.90-1.16) | 1.11 (0.99-1.24) | 1.27 (1.07-1.50) | 1.46 (1.28-1.67) |
| **Vaccination status** | Not vaccinated | 1.00 | 1.00 | 1.00 | 1.00 |
|  | Not recently vaccinated | n.a. | n.a. | 0.68 (0.27-1.70) | n.a. |
|  | Recently vaccinated | 0.42 (0.28-0.62) | 0.48 (0.29-0.79) | 0.52 (0.47-0.57) | 0.58 (0.53-0.64) |
|  |  | **Delta period** | | **Omicron period** | |
|  |  | **Females**  **RR (95% CI)** | **Males**  **RR (95% CI)** | **Females**  **RR (95% CI)** | **Males**  **RR (95% CI)** |
| **Age groups** | 18-39 | 1.00 | 1.00 | 1.00 | 1.00 |
|  | 40-49 | 1.29 (1.11-1.50) | 2.91 (2.52-3.37) | 0.60 (0.52-0.68) | 1.75 (1.48-2.06) |
|  | 50-59 | 2.74 (2.40-3.12) | 5.17 (4.52-5.91) | 0.87 (0.77-0.98) | 3.92 (3.39-4.54) |
|  | 60-69 | 4.73 (4.15-5.39) | 10.08 (8.80-11.55) | 1.90 (1.69-2.13) | 8.05 (6.91-9.37) |
|  | 70-79 | 7.78 (6.75-8.96) | 13.76 (11.83-16.01) | 3.26 (2.86-3.72) | 11.07 (9.36-13.10) |
|  | ≥80 | 9.95 (8.47-11.69) | 17.64 (14.98-20.77) | 4.20 (3.64-4.84) | 12.7 (10.69-15.09) |
| **Citizenship** | LMPC and Italians | 1.00 | 1.00 | 1.00 | 1.00 |
|  | HMPC | 2.46 (2.22-2.71) | 2.02 (1.81-2.27) | 1.94 (1.76-2.14) | 1.60 (1.41-1.82) |
| **Deprivation index - population quintiles** | 1- Least deprived | 1.00 | 1.00 | 1.00 | 1.00 |
|  | 2 | 1.07 (0.97-1.17) | 1.02 (0.94-1.12) | 1.01 (0.93-1.10) | 1.00 (0.92-1.09) |
|  | 3 | 1.01 (0.92-1.11) | 0.98 (0.90-1.07) | 1.00 (0.92-1.09) | 1.06 (0.97-1.15) |
|  | 4 | 1.06 (0.96-1.17) | 1.06 (0.97-1.16) | 0.99 (0.91-1.08) | 1.04 (0.96-1.13) |
|  | 5- Most deprived | 1.01 (0.92-1.11) | 1.06 (0.97-1.16) | 1.05 (0.97-1.14) | 1.11 (1.03-1.21) |
|  | Missing | 1.26 (1.06-1.50) | 1.13 (0.95-1.34) | 1.02 (0.88-1.18) | 1.08 (0.93-1.25) |
| **Risk of hospitalization or death score** | Low | 1.00 | 1.00 | 1.00 | 1.00 |
|  | Moderate | 2.23 (2.00-2.49) | 1.81 (1.64-2.00) | 3.97 (3.55-4.46) | 3.27 (2.93-3.64) |
|  | High | 3.80 (3.30-4.37) | 3.04 (2.68-3.43) | 7.34 (6.41-8.40) | 7.17 (6.35-8.09) |
|  | Very high | 5.40 (4.72-6.19) | 4.39 (3.89-4.94) | 9.18 (8.03-10.5) | 11.00 (9.76-12.39) |
|  | Missing | 1.26 (1.00-1.60) | 1.52 (1.26-1.83) | 1.39 (1.11-1.74) | 1.92 (1.54-2.40) |
| **Vaccination status** | Not vaccinated | 1.00 | 1.00 | 1.00 | 1.00 |
|  | Not recently vaccinated | 0.37 (0.34-0.39) | 0.40 (0.37-0.42) | 0.58 (0.54-0.62) | 0.69 (0.64-0.74) |
|  | Recently vaccinated | 0.30 (0.28-0.33) | 0.31 (0.28-0.34) | 0.53 (0.50-0.57) | 0.52 (0.49-0.56) |

Notes: RR = risk ratio; CI = confidence interval; HMPCs = high migratory pressure countries; LMPCs = low migratory pressure countries; n.a.= not available due to too few or absent events..

**Supplementary Table 5**. Association between explanatory variables and risk of severe disease, by sex and time period.Emilia-Romagna region, February 2020 - February 2022.

|  |  | **Pre-alpha period** | | **Alpha period** | |
| --- | --- | --- | --- | --- | --- |
|  |  | **Females**  **RR (95% CI)** | **Males**  **RR (95% CI)** | **Females**  **RR (95% CI)** | **Males**  **RR (95% CI)** |
| **Age groups** | 18-39 | 1.00 | 1.00 | 1.00 | 1.00 |
|  | 40-49 | 4.30 (3.44-5.39) | 5.43 (4.65-6.34) | 3.31 (2.7-4.07) | 4.58 (3.94-5.32) |
|  | 50-59 | 9.58 (7.77-11.8) | 10.69 (9.25-12.36) | 7.63 (6.32-9.2) | 8.87 (7.71-10.20) |
|  | 60-69 | 22.21 (18.09-27.27) | 20.68 (17.91-23.89) | 16.67 (13.88-20.00) | 15.76 (13.69-18.13) |
|  | 70-79 | 34.52 (28.00-42.57) | 28.15 (24.27-32.66) | 24.21 (20.07-29.21) | 22.14 (19.13-25.63) |
|  | ≥80 | 29.28 (23.62-36.30) | 29.22 (25.11-34.00) | 24.72 (20.27-30.14) | 23.69 (20.31-27.63) |
| **Citizenship** | LMPC and Italians | 1.00 | 1.00 | 1.00 | 1.00 |
|  | HMPC | 1.42 (1.28-1.57) | 1.08 (0.98-1.18) | 1.53 (1.38-1.69) | 1.23 (1.12-1.36) |
| **Deprivation index - population quintiles** | 1- Least deprived | 1.00 | 1.00 | 1.00 | 1.00 |
|  | 2 | 0.99 (0.92-1.06) | 1.05 (1.00-1.11) | 1.03 (0.94-1.12) | 0.97 (0.91-1.04) |
|  | 3 | 1.01 (0.94-1.08) | 1.01 (0.96-1.07) | 1.01 (0.93-1.11) | 1.02 (0.95-1.09) |
|  | 4 | 0.99 (0.93-1.06) | 1.02 (0.97-1.07) | 1.05 (0.96-1.15) | 1.05 (0.98-1.12) |
|  | 5- Most deprived | 1.00 (0.94-1.07) | 1.01 (0.96-1.06) | 1.17 (1.07-1.27) | 1.05 (0.98-1.12) |
|  | Missing | 0.76 (0.66-0.87) | 0.92 (0.83-1.02) | 1.08 (0.91-1.27) | 1.12 (0.99-1.27) |
| **Risk of hospitalization or death score** | Low | 1.00 | 1.00 | 1.00 | 1.00 |
|  | Moderate | 1.89 (1.75-2.05) | 1.46 (1.38-1.54) | 1.72 (1.58-1.87) | 1.38 (1.29-1.47) |
|  | High | 2.25 (2.05-2.47) | 1.74 (1.62-1.87) | 2.22 (1.98-2.49) | 1.75 (1.60-1.91) |
|  | Very high | 2.38 (2.17-2.61) | 1.87 (1.75-2.00) | 2.55 (2.28-2.85) | 2.08 (1.90-2.27) |
|  | Missing | 0.96 (0.79-1.17) | 1.07 (0.92-1.24) | 1.04 (0.81-1.33) | 1.27 (1.07-1.51) |
| **Vaccination status** | Not vaccinated | 1.00 | 1.00 | 1.00 | 1.00 |
|  | Not recently vaccinated | n.a. | n.a. | 0.54 (0.11-2.68) | n.a. |
|  | Recently vaccinated | 0.51 (0.31-0.84) | 0.49 (0.26-0.92) | 0.43 (0.38-0.50) | 0.49 (0.43-0.55) |
|  |  | **Delta period** | | **Omicron period** | |
|  |  | **Females**  **RR (95% CI)** | **Males**  **RR (95% CI)** | **Females**  **RR (95% CI)** | **Males**  **RR (95% CI)** |
| **Age groups** | 18-39 | 1.00 | 1.00 | 1.00 | 1.00 |
|  | 40-49 | 4.07 (3.05-5.43) | 4.27 (3.46-5.26) | 5.32 (2.81-10.09) | 4.81 (2.98-7.77) |
|  | 50-59 | 10.02 (7.67-13.1) | 8.20 (6.75-9.97) | 14.18 (7.77-25.88) | 15.57 (10.04-24.13) |
|  | 60-69 | 19.55 (14.98-25.52) | 17.83 (14.69-21.64) | 39.32 (21.71-71.19) | 44.84 (28.96-69.43) |
|  | 70-79 | 31.64 (23.95-41.80) | 26.47 (21.46-32.66) | 74.00 (40.18-136.29) | 75.73 (48.12-119.18) |
|  | ≥80 | 35.97 (26.53-48.78) | 33.06 (26.29-41.58) | 102.88 (55.00-192.44) | 94.81 (59.78-150.39) |
| **Citizenship** | LMPC and Italians | 1.00 | 1.00 | 1.00 | 1.00 |
|  | HMPC | 2.42 (2.09-2.80) | 1.95 (1.68-2.27) | 1.26 (0.97-1.64) | 1.21 (0.93-1.56) |
| **Deprivation index - population quintiles** | 1- Least deprived | 1.00 | 1.00 | 1.00 | 1.00 |
|  | 2 | 1.21 (1.05-1.39) | 1.08 (0.97-1.22) | 0.92 (0.78-1.07) | 0.94 (0.82-1.08) |
|  | 3 | 1.11 (0.96-1.28) | 0.97 (0.87-1.09) | 0.91 (0.77-1.06) | 1 (0.88-1.15) |
|  | 4 | 1.11 (0.96-1.29) | 1.07 (0.95-1.20) | 0.90 (0.76-1.05) | 0.98 (0.85-1.13) |
|  | 5- Most deprived | 1.13 (0.98-1.30) | 1.05 (0.94-1.18) | 1.02 (0.87-1.18) | 1.1 (0.96-1.26) |
|  | Missing | 1.12 (0.85-1.48) | 1.10 (0.88-1.38) | 0.81 (0.59-1.11) | 1.05 (0.82-1.36) |
| **Risk of hospitalization or death score** | Low | 1.00 | 1.00 | 1.00 | 1.00 |
|  | Moderate | 2.03 (1.75-2.35) | 1.47 (1.30-1.66) | 4.27 (3.41-5.35) | 2.62 (2.20-3.11) |
|  | High | 3.43 (2.81-4.20) | 2.45 (2.09-2.87) | 8.82 (6.79-11.45) | 6.01 (4.95-7.30) |
|  | Very high | 5.19 (4.30-6.27) | 3.40 (2.90-3.98) | 13.25 (10.26-17.11) | 9.71 (8-11.78.00) |
|  | Missing | 1.33 (0.95-1.86) | 1.08 (0.82-1.42) | 2.81 (1.78-4.42) | 2.54 (1.79-3.6) |
| **Vaccination status** | Not vaccinated | 1.00 | 1.00 | 1.00 | 1.00 |
|  | Not recently vaccinated | 0.25 (0.22-0.27) | 0.3 (0.28-0.33) | 0.46 (0.4-0.52) | 0.49 (0.44-0.54) |
|  | Recently vaccinated | 0.16 (0.14-0.19) | 0.2 (0.18-0.23) | 0.22 (0.2-0.25) | 0.24 (0.22-0.27) |

Notes: RR = risk ratio; CI = confidence interval; HMPCs = high migratory pressure countries; LMPCs = low migratory pressure countries; n.a.= not available due to too few or absent events.

**Supplementary Table 6**. Association between explanatory variables and risk of death, by sex and time period. Emilia-Romagna region, February 2020 - February 2022.

|  |  | **Pre-alpha period** | | **Alpha period** | |
| --- | --- | --- | --- | --- | --- |
|  |  | **Females**  **RR (95% CI)** | **Males**  **RR (95% CI)** | **Females**  **RR (95% CI)** | **Males**  **RR (95% CI)** |
| **Age groups** | 18-39 | 1.00 | 1.00 | 1.00 | 1.00 |
|  | 40-49 | 2.77 (1.12-6.84) | 5.09 (2.41-10.75) | 5.20 (1.08-25.07) | 8.5 (2.94-24.57) |
|  | 50-59 | 11.00 (5.04-24.01) | 19.50 (9.90-38.39) | 18.40 (4.33-78.18) | 19.29 (6.98-53.26) |
|  | 60-69 | 41.37 (19.34-88.49) | 75.18 (38.58-146.53) | 69.16 (16.74-285.74) | 64.03 (23.27-176.22) |
|  | 70-79 | 92.64 (42.97-199.72) | 157.51 (80.49-308.25) | 144.04 (34.53-600.82) | 154.58 (55.4-431.33) |
|  | ≥80 | 143.61 (66.42-310.52) | 242.11 (123.58-474.33) | 247.5 (58.79-1042.02) | 261.03 (93.03-732.46) |
| **Citizenship** | LMPC and Italians | 1.00 | 1.00 | 1.00 | 1.00 |
|  | HMPC | 0.61 (0.45-0.84) | 0.84 (0.66-1.07) | 0.79 (0.50-1.24) | 0.97 (0.69-1.37) |
| **Deprivation index - population quintiles** | 1- Least deprived | 1.00 | 1.00 | 1.00 | 1.00 |
|  | 2 | 1.02 (0.93-1.11) | 0.98 (0.91-1.05) | 1.01 (0.84-1.20) | 0.92 (0.79-1.07) |
|  | 3 | 0.97 (0.89-1.06) | 0.92 (0.85-0.99) | 1.01 (0.84-1.20) | 1.02 (0.89-1.18) |
|  | 4 | 0.99 (0.91-1.08) | 1.02 (0.95-1.09) | 1.04 (0.87-1.24) | 1.05 (0.91-1.21) |
|  | 5- Most deprived | 1.07 (0.98-1.16) | 0.98 (0.91-1.05) | 1.16 (0.98-1.37) | 1.1 (0.96-1.27) |
|  | Missing | 0.88 (0.75-1.03) | 0.93 (0.81-1.07) | 0.86 (0.60-1.23) | 1.06 (0.82-1.37) |
| **Risk of hospitalization or death score** | Low | 1.00 | 1.00 | 1.00 | 1.00 |
|  | Moderate | 3.47 (2.88-4.19) | 2.42 (2.11-2.77) | 3.96 (2.99-5.24) | 2.94 (2.3-3.74) |
|  | High | 5.72 (4.71-6.96) | 4.20 (3.63-4.86) | 8.95 (6.61-12.1) | 6.25 (4.82-8.12) |
|  | Very high | 7.88 (6.50-9.55) | 5.60 (4.86-6.46) | 14.28 (10.61-19.23) | 9.33 (7.2-12.07) |
|  | Missing | 4.70 (3.70-5.97) | 2.87 (2.26-3.65) | 5.91 (3.46-10.09) | 3.7 (2.26-6.05) |
| **Vaccination status** | Not vaccinated | 1.00 | 1.00 | 1.00 | 1.00 |
|  | Not recently vaccinated | n.a. | n.a. | n.a. | n.a. |
|  | Recently vaccinated | 0.67 (0.43-1.04) | 0.46 (0.20-1.01) | 0.53 (0.45-0.64) | 0.5 (0.42-0.61) |
|  |  | **Delta period** | | **Omicron period** | |
|  |  | **Females**  **RR (95% CI)** | **Males**  **RR (95% CI)** | **Females**  **RR (95% CI)** | **Males**  **RR (95% CI)** |
| **Age groups** | 18-39 | 1.00 | 1.00 | 1.00* | 1.00 |
|  | 40-49 | 5.21 (0.54-50.32) | 2.98 (0.85-10.48) |  | 1.04 (0.17-6.21) |
|  | 50-59 | 35.2 (4.63-267.68) | 16.61 (5.94-46.49) |  | 14.25 (4.29-47.38) |
|  | 60-69 | 97.89 (13.2-726.14) | 44.09 (15.75-123.40) | 10.89 (4.97-23.84) | 38.96 (11.62-130.64) |
|  | 70-79 | 242.51 (32.59-1804.55) | 118.82 (41.27-342.11) | 43.55 (19.05-99.59) | 102.97 (29.92-354.41) |
|  | ≥80 | 382.05 (50.90-2867.45) | 236.07 (80.81-689.66) | 126.81 (52.82-304.46) | 209.14 (60.11-727.68) |
| **Citizenship** | LMPC and Italians | 1.00 | 1.00 | 1.00 | 1.00 |
|  | HMPC | 1.66 (1.04-2.66) | 0.76 (0.38-1.52) | 0.6 (0.29-1.23) | 0.65 (0.32-1.34) |
| **Deprivation index - population quintiles** | 1- Least deprived | 1.00 | 1.00 | 1.00 | 1.00 |
|  | 2 | 1.23 (0.96-1.57) | 0.92 (0.74-1.15) | 0.77 (0.61-0.98) | 0.81 (0.65-1.01) |
|  | 3 | 0.92 (0.71-1.19) | 0.99 (0.80-1.22) | 0.76 (0.59-0.97) | 0.91 (0.74-1.13) |
|  | 4 | 1.06 (0.82-1.37) | 1.03 (0.83-1.28) | 0.81 (0.64-1.03) | 0.78 (0.62-0.98) |
|  | 5- Most deprived | 0.93 (0.72-1.20) | 1.02 (0.82-1.27) | 0.98 (0.79-1.22) | 0.89 (0.72-1.11) |
|  | Missing | 1.35 (0.83-2.20) | 1.02 (0.66-1.57) | 0.77 (0.49-1.21) | 0.90 (0.59-1.37) |
| **Risk of hospitalization or death score** | Low | 1.00 | 1.00 | 1.00 | 1.00 |
|  | Moderate | 3.92 (2.48-6.19) | 2.19 (1.51-3.17) | 4.02 (2.24-7.22) | 4.75 (3.05-7.39) |
|  | High | 12.9 (7.94-20.98) | 6.20 (4.21-9.13) | 10.71 (5.8-19.79) | 13.16 (8.24-21.01) |
|  | Very high | 23.72 (14.76-38.14) | 11.62 (7.96-16.96) | 18.95 (10.35-34.68) | 24.23 (15.28-38.44) |
|  | Missing | 6.10 (2.89-12.85) | 6.39 (3.6-011.33) | 9.85 (4.97-19.50) | 6.82 (3.48-13.35) |
| **Vaccination status** | Not vaccinated | 1.00 | 1.00 | 1.00 | 1.00 |
|  | Not recently vaccinated | 0.42 (0.35-0.50) | 0.55 (0.48-0.64) | 0.64 (0.52-0.78) | 0.65 (0.54-0.78) |
|  | Recently vaccinated | 0.25 (0.19-0.33) | 0.32 (0.26-0.40) | 0.26 (0.22-0.31) | 0.24 (0.20-0.29) |

Notes: RR = risk ratio; CI = confidence interval; HMPCs = high migratory pressure countries; LMPCs = low migratory pressure countries; n.a.= not available due to too few or absent events; * = due to the too small number of events, the first three age classes were reclassified into a single reference class (18-59 years).

**Supplementary Table 7**. Risk ratio of hospitalization, disease severity and mortality among HMPCs residents, by time of residency. Emilia-Romagna region, February 2020 - February 2022.

| **Standard age class model** | | | | | | | | | | | | |
| --- | --- | --- | --- | --- | --- | --- | --- | --- | --- | --- | --- | --- |
|  | **Hospitalization** | | | | **Severe disease** | | | | **Death** | | | |
|  | **Males** | | **Females** | | **Males** | | **Females** | | **Males** | | **Females** | |
|  | **RR (95% CI)** |  | **RR (95% CI)** |  | **RR (95% CI)** |  | **RR (95% CI)** |  | **RR (95% CI)** |  | **RR (95% CI)** |  |
| **LMPCs** | 1.00 |  | 1.00 |  | 1.00 |  | 1.00 |  | 1.00 |  | 1.00 |  |
| **HMPCs 1-5** | 1.87 (1.71-2.05) | ** | 2.44 (2.26-2.63) | ** | 1.32 (1.14-1.51) | ** | 1.79 (1.57-2.04) | ** | 0.59 (0.36-0.96) | * | 0.48 (0.27-0.83) | ** |
| **HMPCs 6-8** | 1.51 (1.40-1.63) | ** | 1.81 (1.69-1.94) | ** | 1.31 (1.17-1.46) | ** | 1.68 (1.51-1.87) | ** | 0.89 (0.65-1.21) |  | 0.84 (0.59-1.19) |  |
| **HMPCs >8** | 1.46 (1.38-1.55) | ** | 1.58 (1.49-1.68) | ** | 1.36 (1.25-1.47) | ** | 1.57 (1.44-1.71) | ** | 0.93 (0.73-1.18) |  | 0.76 (0.57-1.01) |  |
| **5-year age class model** | | | | | | | | | | | | |
|  | **Hospitalization** | | | | **Severe disease** | | | | **Death** | | | |
|  | **Males** | | **Females** | | **Males** | | **Females** | | **Males** | | **Females** | |
|  | **RR (95% CI)** |  | **RR (95% CI)** |  | **RR (95% CI)** |  | **RR (95% CI)** |  | **RR (95% CI)** |  | **RR (95% CI)** |  |
| **LMPCs** | 1.00 |  | 1.00 |  | 1.00 |  | 1.00 |  | 1.00 |  | 1.00 |  |
| **HMPCs 1-5** | 1.92 (1.76-2.10) | ** | 2.40 (2.23-2.59) | ** | 1.35 (1.17-1.55) | ** | 1.81 (1.59-2.07) | ** | 0.61 (0.38-0.99) | * | 0.51 (0.29-0.89) | * |
| **HMPCs 6-8** | 1.52 (1.41-1.64) | ** | 1.78 (1.66-1.90) | ** | 1.32 (1.18-1.47) | ** | 1.69 (1.52-1.88) | ** | 0.92 (0.68-1.27) |  | 0.90 (0.63-1.27) |  |
| **HMPCs >8** | 1.48 (1.39-1.56) | ** | 1.57 (1.48-1.66) | ** | 1.37 (1.26-1.48) | ** | 1.58 (1.45-1.72) | ** | 0.95 (0.75-1.22) |  | 0.81 (0.61-1.07) |  |

Notes: HMPCs = high migratory pressure countries; LMPCs = low migratory pressure countries; RR = risk ratio; CI = confidence interval; * = p < 0.05; ** = p < 0.01. Results are presented for two different regression models, considering standard and 5-year age classes respectively.
